# Supplementary material for: Pharmacokinetic Imaging Using 99mTc-Mebrofenin to Untangle the Pattern of Hepatocyte Transporter Disruptions Induced by Endotoxemia in Rats
Source: Pharmaceuticals (Basel). 2022 Mar 24;15(4):392. doi: 10.3390/ph15040392 (PMC9028474; doi:10.3390/ph15040392)
Supplement: Supplementary file 1 [file pharmaceuticals-15-00392-s001.zip › pharmaceuticals-1616596-supplementary.pdf]

Supplementary Materials

Table S1. Expression levels of transporter mRNA of selected genes in the liver of control and LPS-treated rats (24h after exposure to LPS).

	**ABC transporters**					
**Transporter (gene)**	**P-gp (*Abcb1a/Mdr1a*)**	**P-gp (*Abcb1b/Mdr1b*)**	**Mrp2 (*Abcc2*)**	**Mrp3 (*Abcc3*)**	**Mrp4 (*Abcc4*)**	
Healthy rats (n = 4)	2.9 ± 0.7	21.6 ± 3.4	129.6 ± 28.7	2.2 ± 0.9	2.7 ± 0.9	
LPS-treated rats (n = 4)	0.9 ± 0.4	5.4 ± 2.4	50.1 ± 20.9	0.8 ± 0.5	1.4 ± 0.6	
	**SLC transporters**					
**Transporter (gene)**	**Ntcp (*Slc10a1*)**	**Oat2 (*Slc22a7*)**	**Oatp1 (*Slco1a1*)**	**Oatp2 (*Slco1a4*)**	**Oatp4 (*Slco1b2*)**	**Oct1 (*Slc22a1*)**
Healthy rats (n = 4)	248.0 ± 26.8	17.0 ± 2.9	74.4 ± 6.8	63.9 ± 8.9	193.5 ± 51.6	49.3 ± 8.6
LPS-treated rats (n = 4)	125.1 ± 17.6	18.7 ± 9.0	39.5 ± 9.8	10.9 ± 7.1	118.8 ± 53.8	22.9 ± 2.8
	**Cytochromes**					
**Cytochrome (gene)**	**Cyb2b1 (*Cyp2b1*)**	**Cyp3a1 (*Cyp3a23-3a1*)**				
Healthy rats (n = 4)	0.4 ± 0.2	158.5 ± 26.7				
LPS-treated rats (n = 4)	0.2 ± 0.1	41.0 ± 8.3				

Values are in arbitrary units. (Abcb: ATP-binding cassette sub-family B, Abcc: ATP-binding cassette sub-family C, Cyp: cytochrome-P450, LPS: lipopolysaccharide, Mdr: multidrug resistance protein, Mrp: multidrug-resistance associated protein, Ntcp: Na+-taurocholate cotransporting polypetide, Oat: organic anion transporter, Oatp: organic anion transporting polypeptide, Oct: organic cation transporter, Slc: Solute carrier family)

Table S2. Area under the time-activity curves (AUC) of ^{99m}Tc-mebrofenin in the blood pool (imaged-derived), liver and intestine obtained in all the studied rat groups.

Group	AUC_{blood} (cps/MBq).min	AUC_{liver} (cps/MBq).min	$AUC_{intestine}$ (cps/MBq).min
**Healthy** (n = 5)	51± 14	461 ± 109	425 ± 85
**RIF 9 mg/kg** (n = 5)	105 ± 23	559 ± 123	237 ± 55
**RIF 40 mg/kg** (n = 5)	109 ± 12	385 ± 28	223 ± 54
**LPS** (n = 6)	129 ± 57	814 ± 164	277 ± 65
**LPS + RIF 9 mg/kg** (n = 6)	159 ± 46	663 ± 188	202 ± 37
**LPS + RIF 40 mg/kg** (n = 6)	154 ± 70	564 ± 43	137 ± 60

Radioactivity is expressed in counts per second (cps) in each region of interest, normalized to the injected radioactivity amount in each animal (cps/MBq). Data are given as mean ± SD. (LPS: lipopolysaccharide, RIF: rifampicin). The data of healthy and RIF 40 mg/kg groups have already been presented in a previous study [29].

Table S3. Transfer rate constants obtained with the four-compartment model describing the hepatobiliary disposition of ^{99m}Tc-mebrofenin in all the studied rat groups.

Group	k_1 (min^{-1})	k_2 (min^{-1})	k_3 (min^{-1})	k_5 (min^{-1})
**Healthy**<br>(n = 5)	7.37 ± 1.62<br>(4.8 - 16)	0.15 ± 0.08<br>(7.2 - 30.8)	0.15 ± 0.08<br>(3.9 - 11.9)	0.12 ± 0.03<br>(2.4 - 12.5)
**RIF 9 mg/kg**<br>(n = 5)	4.16 ± 1.43<br>(1.5 - 10.4)	0.04 ± 0.02<br>(4.0 - 17.6)	0.02 ± 0.01<br>(1.6 - 5.2)	1.04 ± 0.62<br>(17.0 - 53.2)
**RIF 40 mg/kg**<br>(n = 5)	0.89 ± 0.09<br>(3.6 - 7.6)	0.01 ± 0.01<br>(28.7 - 125.7)	0.05 ± 0.02<br>(2.4 - 12.7)	0.64 ± 0.88<br>(13.6 - 31.7)
**LPS**<br>(n = 6)	8.72 ± 3.37<br>(1.6 - 9.9)	0.07 ± 0.03<br>(2.9 - 17.8)	0.02 ± 0.01<br>(1.9 - 35.3)	0.30 ± 0.39<br>(9.8 - 49.3)
**LPS + RIF 9 mg/kg**<br>(n = 6)	5.14 ± 3.05<br>(2.2 - 13.1)	0.08 ± 0.04<br>(4.0 - 19.4)	0.05 ± 0.02<br>(2.0 - 14.5)	0.08 ± 0.09<br>(6.6 - 19.4)
**LPS + RIF 40 mg/kg**<br>(n = 6)	1.9 ± 0.78<br>(1.0 - 6.4)	0.03 ± 0.03<br>(7.2 - 39.9)	0.03 ± 0.01<br>(3.0 - 61.4)	0.10 ± 0.10<br>(14.7 - 69.9)

Values are given as mean ± SD. Values in parentheses express percent coefficient of variation (%CV) of the parameters (parameter precision). k_1 and k_2 are the parameters describing the transfer of radiotracer between blood and liver compartments, k_3 describes the radiotracer transfer from liver tissue to the intrahepatic bile ducts and k_5 describes the transfer of radiotracer from the intrahepatic bile ducts to the intestines (excreted bile). The data of healthy and RIF 40 mg/kg groups have already been presented in a previous study [29].

Table S4. Primer sequences used for qPCR assays.

Gene (Protein)	Forward Primer (5′-3′)	Reverse Primer (5′-3′)
Rn 18S	CGCCGCTAGAGGTGAAATTC	TTGGCAAATGCTTTCGCTC
Abcb1a/Mdr1a **(P-gp)**	CAGGTTGGCTGGACAGATTT	CATGTCTCCAAAGACCAGCA
Abcb1b/Mdr1b **(P-gp)**	TTTGACTCGGGAGCAGAAGT	AAGGTGATCCCAAAGACGTG
Abcc2 **(Mrp2)**	CGTCTCCTACGGTTTCCAGA	ACTCAGAAAGGAAGCCGTGA
Abcc3 **(Mrp3)**	AGATCGCAGAGACAGGCAAT	CCAGCATACAGGAGGCAGAT
Abcc4 **(Mrp4)**	TCAGATTGGTTATGAGGTCG	CCATTCTGGTTATTCTGCTG
Slc10a1 **(Ntcp)**	CAAGTCCAAAAGGCCACACT	TGATGACAGAGAGGGCTGTG
Slc22a7 **(Oat2)**	CGCTCAGAATTCTCCTCCAC	GCACACCAATGAAGAAGCAG
Slco1a1 **(Oatp1)**	AGGTCGAGAATGACGGAGAA	ATTCCGAGGAAGGGAAGTGT
Slco1a4 **(Oatp2)**	TGGGATCTGTTTTCCACACA	ATCTCGCTACCCCACCATTTCCT
Slco1b2 **(Oatp4)**	GACATCACCCACTGGACCTT	AGCATGTTCCCCATCAAGAC
Slc22a1 **(Oct1)**	GGTTGCCAACAGGAGTCAGT	AGACAGAGCTTACGGCCAAA
Cyp2b1 **(Cyp2b1)**	GGCTCACACCGGCTACCAA	TGAAAACCTCTGAATCTCGTGGATA
Cyp3a23-3a1 **(Cyp3a1)**	GTAAAATACTTGAGGCAAGAGAAAGGC	TCGGGTTGTTGAGGGAATCA

(Abcb: ATP-binding cassette sub-family B, Abcc: ATP-binding cassette sub-family C, Cyp: cytochrome-P450, Mdr: multidrug resistance protein, Mrp: multidrug-resistance associated protein, Ntcp: Na+-taurocholate cotransporting polypetide, Oat: organic anion transporter, Oatp: organic anion transporting polypeptide, Oct: organic cation transporter, Slc: Solute carrier family)

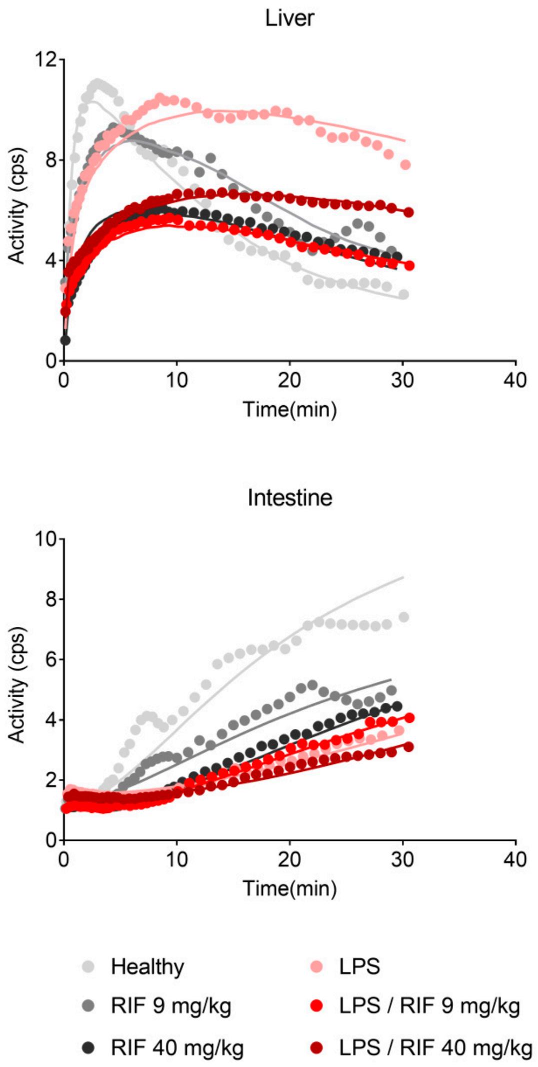

Figure S1. Time-activity curves of ^{99m}Tc-mebrofenin in the liver and intestine for observed data (circles) and model fits (solid lines) in a representative subject of each studied rat group. (LPS: lipopolysaccharide, RIF: rifampicin). The data of healthy and RIF 40 mg/kg groups have already been presented in a previous study [29].
